# 333. Comparing COVID-19-related Morbidity and Mortality between Patients with and without Substance Use Disorder: A Retrospective Cohort Study

**DOI:** 10.1093/ofid/ofab466.534

**Published:** 2021-12-04

**Authors:** Angela McLaughlin, Rebecca Burns, Morgan Ryan, Sabrina A Assoumou

**Affiliations:** 1 Boston Medical Center, Boston, Massachusetts; 2 Boston University School of Medicine, Boston, Massachusetts; 3 Boston University School of Public Health, Boston, Massachusetts; 4 Boston University, Boston, MA

## Abstract

**Background:**

Early data suggest that people with substance use disorder (SUD) who develop coronavirus disease 2019 (COVID-19) have increased intubation and mortality rates when compared to those without SUD. Information on other COVID-19-related complications in this population is limited. We evaluated COVID-19 outcomes in patients with and without SUD.

**Methods:**

We created a retrospective cohort of patients with COVID-19 admitted to an urban safety net hospital from 3/16/2020 to 4/8/2020. Inclusion criteria were admission with laboratory-confirmed severe acute respiratory syndrome coronavirus 2 and age greater than 18 years. SUD included alcohol use disorder or heavy alcohol use as defined by the National Institute on Alcohol Abuse and Alcoholism, use of cocaine, non-prescribed opioids or amphetamines. Primary outcome was inpatient mortality. Secondary outcomes were clinical complications (intubation, secondary infections, renal failure, venous thromboembolism, stroke, hepatitis, myocardial infarct, multisystem organ failure) and resource utilization (length of stay, intensive care unit [ICU] admission, ICU days, readmission). We used multivariable regression to assess factors associated with mortality and length of stay, and univariate analyses for other outcomes.

**Results:**

Of 409 included patients, 70 (17.1%) had SUD. Those with SUD were more likely to be male and have pulmonary disease or hepatitis C. There were no differences in other comorbidities, mean age or race/ethnicity. After multivariable analysis, SUD was not associated with mortality (aOR 1.60; 95% CI, 0.60-3.81). Similarly baseline oxygenation defined as the ratio of oxygen saturation to fraction of inspired oxygen (aOR 1.57; 0.11-13.0) and administration of immunomodulatory therapy (tocilizumab, sarilumab or anakinra) (aOR 1.41; 0.65-3.01) did not affect mortality. In contrast, age (aOR 1.06; 1.03-1.09), sex (aOR 2.30; 1.04-5.47) and obstructive sleep apnea (aOR 4.07; 1.64-9.66) were associated with mortality. We did not find any associations with secondary outcomes.

**Conclusion:**

Our findings suggest that substance use alone may not increase COVID-19 adverse outcomes. Future studies should evaluate these results in the current period of improved COVID-19 therapy.

**Disclosures:**

**All Authors**: No reported disclosures

